# Clinical Identification of Hypovitaminosis D among Elderly Attending Primary Care Centre in Saudi Arabia

**DOI:** 10.1155/2022/6341645

**Published:** 2022-10-12

**Authors:** Abdullah M. Alzahrani, Leena S. Emam, Maryam S. Alsharif, Alqassem Y. Hakami, Syed Sameer Aga

**Affiliations:** ^1^Department of Primary Health Care, Ministry of National Guard Health Affairs (MNGHA), King Abdulaziz Medical City, Jeddah, Saudi Arabia; ^2^Research Office, King Abdullah International Medical Research Centre (KAIMRC), Ministry of National Guard Health Affairs (MNGHA), King Abdulaziz Medical City, Jeddah, Saudi Arabia; ^3^Department of Primary Health Care, Ministry of Health (MOH), Jeddah, Saudi Arabia; ^4^Department of Basic Medical Sciences, College of Medicine, King Saud Bin Abdul Aziz University for Health Sciences (KSAU-HS), King Abdulaziz Medical City, Jeddah, Saudi Arabia

## Abstract

**Background:**

A large proportion of elderly people suffer from hypovitaminosis D, and depending on the severity of the condition, they develop complications that are detrimental to their health.

**Objective:**

To determine the consistency between the results of the vitamin D level in the blood compared to the result with the score of a simple questionnaire (Physician Vitamin D Status Predictor - VDSP) for elderly patients. *Subjects and methods.* This is a cross-sectional study conducted during the period between October 2018 and November 2019 in 3 primary health care centres (PHCCs) in Jeddah, Saudi Arabia. The subjects for this study were patients aged 60 or older. Data were collected in two phases: a questionnaire approach as the first phase, while the second phase involved blood testing for vitamin D levels. The validated questionnaire used in this study was the Physician`s Vitamin D Status Predictor (VDSP).

**Results:**

The study included 335 participants who ranged between 60 and 107 years old with a mean age of 68.2 years and standard deviation (SD) of 7.3 years. Females represented 66.9% of the total participants. The prevalence of vitamin D deficiency was 60.8%; among them, 7.2% were categorized as severe deficiency, whereas the prevalence of vitamin D insufficiency was 29.9%. The outcomes of VDSP survey were not directly associated with serum 25(OH)D levels in elderly people, except for the number of medications. In addition, vitamin D supplementation was associated with serum 25(OH)D levels among those patients.

**Conclusion:**

Vitamin D supplementation was significantly associated with serum 25(OH)D levels. Moreover, this study showed a significant association between serum 25(OH)D levels and the number of medications taken by the participants.

## 1. Introduction

Hypovitaminosis D is highly prevalent in elderly people and associated with several known adverse effects [1]. According to a study conducted in Saudi Arabia, the prevalence of vitamin D deficiency in general population was over 80% [2]. Comparatively, a study conducted on Singapore population revealed a prevalence of around 85.6% [3]. Importantly, Middle East countries are among the highest in the prevalence of hypovitaminosis D worldwide [2]. Precise to the elderly population, Annweiler [4] reported that over 90% of elderly people in Europe and the United States were affected with hypovitaminosis D. In Italy, hypovitaminosis D has been found to have a prevalence of 76% in elderly females with 25(OH)D concentration below 12 ng/mL [5].

Hypovitaminosis D affects both developing as well as developed countries. Its risk factors are similar regardless of geographical location or status of the country. Generally, the risk factors include old age, gender (more in women), seasons (higher in the winter), dark skin pigmentation, malnutrition, deficiency of sunlight, obesity, and covered clothing style [6]. Old age is an independent risk factor for this condition, and it may, depending on the severity of the condition, develop complications that are detrimental to their health. In the elderly, Huang et al. [7] reported that inadequate intake of vitamin D-rich food and inadequate sun exposure are the major risk factors for hypovitaminosis D. Isaia et al. [5] in their study found that age, the number of pregnancies, smoking, and physical inactivity increased the probability of developing hypovitaminosis D. Importantly, vitamin D plays a vital role in the elderly, and traditionally, it has been used as one of the major contributing factors to maintain the bone health of old people [8]. Vitamin D is known to help aging patients in preventing as well as managing old-age diseases such as low cognition, depression, hypertension, osteoporosis, and cardiovascular diseases [9]. Bruyere et al. [10] revealed that vitamin D may reduce falls and prevents bone fractures and muscle weaknesses. These findings are supported by Sahota [11] who reported vitamin D significantly reduces fall and muscle weakness by promoting physical exercise and fitness. Vitamin D deficiency is also associated with secondary hyperparathyroidism, bone loss, the risk of developing osteoporosis [2], and in the development of sarcopenia [3]. Annweiler et al. [4] reported an association between hypovitaminosis D and dysfunction of target tissues, leading to cancer, *tuberculosis*, hypertension, multiple sclerosis, depression, dementia, and propensity to fall. With such complications, it is important to emphasise on the process of screening and diagnosis of the disorder. In addition, it is crucial to understand that vitamin D screening is not a global usual practice due to the high cost [12]. Research, however, has recommended the necessity of screening in severe deficiency only [12]. Diagnosis recommendation includes measuring the following: the total 25(OH)D level in the blood after ingestion, the level of cutaneous, and serum vitamin D converted into 25(OH)D [12].

Despite the recommendations of using blood tests as a requirement to determine the severity of the deficiency, most health practitioners start the supplement use directly after the diagnosis [1]. While blood tests are highly utilized in elderly people, the technique is extremely expensive. A recent study from France reported that blood testing for vitamin D in elderly was not routinely recommended due to the lack of clinical use evidence for the serum 25(OH)D test [13]. In such cases, supplementation with appropriate blood tests is recommended to adjust the doses of vitamin D in order to avoid any side effects, such as fall or allergies in elderly patients [13].

In consideration, there is emerging evidence demonstrating the importance to identify vitamin D status in older people without the need for blood testing. Thus, there is an urgent need to establish a new technique in detecting this deficiency, for example, a simple questionnaire that can adequately detect hypovitaminosis D, leading to a cost-effective approach in diagnosing and treating this critical age group. In this study, we examined whether a recently developed simple tool (the Vitamin D Status Predictor VDSP) can consistently identify elderly patients with undesirable vitamin D status compared to blood testing. If the results of the score from the questionnaires are consistent with the lab outcomes, then it would be a cost-effective approach to use the tool as a way of measuring vitamin D levels in elderly to prevent complications of hypovitaminosis D. Moreover, applying this questionnaire approach might lead to less time and resources necessary for the diagnosis. For instance, patients will not be required to visit the clinic again for follow-up on the lab results, and treatment can be started as early as possible. The aim of the study was to determine whether the levels of serum vitamin D were consistent with the results obtained by employing the physician VDSP.

## 2. Subjects and Methods

This is a cross-sectional study conducted during the period between October 2018 and November 2019 in 3 primary health care centres (PHCCs), including the specialized polyclinics, AlWaha, Iskan, and Bahra at National Guard Hospital in Jeddah, Saudi Arabia. These polyclinics are under the Clinical Nutrition Department of the Ministry of National Guard-Health Affairs (MNG-HA). The reason behind choosing PHCC in this study is they provide primary care coverage of preventive medicine and nutritional health to all age categories [14].

According to a 2016 report, the population of elderly people in Saudi Arabia is estimated at 1,752,949 [15]. The latter study included participants aged 60 or older based on the definition of elderly in Saudi Arabia. The selection of participants to include in the study was based on the following inclusion criteria: (1) elderly patients aged 60 or older, (2) attending family medicine clinics at the selected PHCCs, (3) eligible to be treated in MNG-HA, and (4) Saudi nationality. The exclusion criteria were (a) age < 60 years and (b) patients on regular vitamin D supplements. The estimated sample was 384 patients, calculated using a 5% precision, with 80% prevalence and a margin of error of ±5%, and with 95% confidence interval. The sample was selected using a convenience nonprobability sampling technique among patients attending the selected PHCCs.

Data were collected in two different phases. For the first phase, a questionnaire was used to collect the required data from the participants. In addition, the second phase included the use of blood testing for vitamin D levels. The questionnaire was adopted from Vitamin D Status Predictor (VDSP), a nonlinear model of feed forward artificial neural network [1]. The physician-VDSP questionnaire developed by Annweiler et al. [13] was utilized to collect the data through an interview session by researchers (family medicine residents). In detail, after obtaining the written consent, the physician filled the VDSP questionnaire during patient visits if they met the eligibility criteria to be assessed for hypovitaminosis D. An additional question regarding the current use of vitamin D supplements was added to the questionnaire in order to determine the type and frequency of supplement use. The questionnaire contains items on variables such as “gait and fall” and “osteoporosis” [13]. Finally, estimates of the vitamin D level were carried out through the collection of 3 ml of venous blood samples for the identification of vitamin D levels. The validity and reliability of the VDSP questionnaires were assessed by Annweiler et al. [13]. Moreover, a pilot study was conducted in the same centres on a similar group of patients (*N* = 20), and the results were included in the present study. In addition, consent was taken from the patient before filling or starting the study as previously mentioned. The study was approved by the Research Ethics Committee of King Abdullah International Medical Research Centre, Jeddah, KSA. In addition, confidentiality of the data was maintained throughout the study.

The demographics of participants were analyzed using frequencies and percentages as well as the means with standard deviation wherever appropriate. Univariate analysis was conducted to identify hypovitaminosis D risk factors (Table1). *p* values <0.05 were considered significant. Analysis was conducted by SPSS (v25.0, IBM Corporation, Chicago, IL, USA).

## 3. Results

The study included 335 participants with age ranging between 60 and 107 with a mean of 68.2 years and standard deviation (SD) of 7.3 years. Importantly, females represented 66.9% of the total participants.

**Figure 1:**
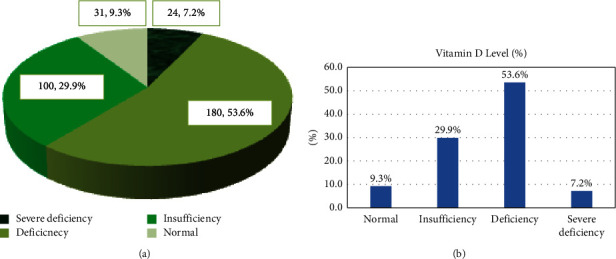
(a): Vitamin D levels among the participants. (b): Vitamin D levels among the participants.

The statistical analysis revealed a prevalence of vitamin D deficiency around 60.9%; moreover, 7.2% showed a severe outcome of deficiency. In addition, the prevalence of vitamin D insufficiency was 29.9% as illustrated in Figures 1(a) and 1(b).

Among several studied factors, only two factors were significantly associated with vitamin D levels as shown in Table 2. Vitamin D insufficiency was more reported among participants on prescription of six or more drugs as compared to participants on less than six drug prescriptions (69% vs. 31%). Importantly, vitamin D deficiency was more prominent among participants on prescription containing six drugs or more daily as compared to prescription of less than six drugs (52.2% vs. 47.8%), *p*=0.010. Statistical analysis revealed that severe deficiency as well as deficiency of vitamin D were more likely to be reported among participants without any vitamin D supplements as compared to participants on vitamin D supplements (83.3% and 66.1% vs. 16.7% and 33.9%, respectively), whereas vitamin D insufficiency was more reported among those who have taken vitamin D supplements compared to those who did not take them (57% vs. 43%), *p* < 0.001. Other studied factors were not significantly associated with vitamin D levels.

**Figure 2:**
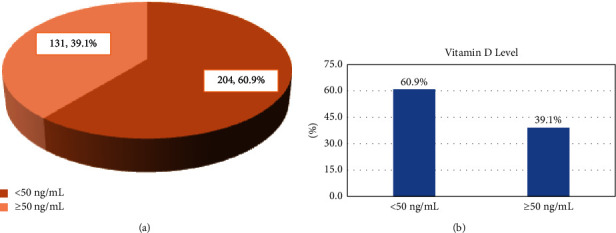
(a): Vitamin D level among the participants. (b): Vitamin D level among the participants.

Figures 2(a) and 2(b) illustrate that 60.9% of the elderly participants had a vitamin D level <50 ng/ml. The vitamin D level <50 ng/ml was more likely to be reported among participants who intake six drugs or more compared to those who intake less than six drugs (52% vs. 48%), *p*=0.001. Vitamin D <50 ng/ml was more reported among participants without any vitamin D supplements as compared to those on vitamin D supplements (68.1 vs. 31.9%), *p* < 0.001. Other studied factors were not significantly associated with vitamin D levels as presented in Table 1.

## 4. Discussion

The present study was conducted to define the variables affecting serum 25OHD concentration in elderly patients using physicians' VDSP tool [16]. In addition, we incorporated another part to measure the intake of vitamin D supplementation.

The primary hypothesis of the present study was to identify vitamin D deficiency/insufficiency in elderly patients, based on physician-reported demographic and previous clinical data, rather than depending on a blood test as evidence. It is proposed that VDSP completed by the elderly persons themselves could be of value in providing information to the physicians, could help to identify those with vitamin D deficiency/insufficiency, and ultimately guide in the management plan. However, in the present study as a result of a high illiteracy rate of the participants and noncooperation of relatives, we used only the physician-reported form of the questionnaire.

In the present study, the prevalence of vitamin D deficiency was 53.6% whereas that of insufficiency was 29.9%. In similar line with our findings, other reports conducted in Brazil and France have reported a high prevalence of vitamin D deficiency in the elderly age group, 66.7% and 70%, respectively [17–19]. In addition, all the studied variables of the 17 items of VDSP were not significantly associated with the vitamin D level. However, statistical analysis revealed a significant association between the number of medications taken per day and vitamin D insufficiency. Vitamin D insufficiency was more reported among participants on six drugs or more daily, whereas vitamin D deficiency was more seen among participants who are taking less than six drugs daily. This finding might be hypothesized by the possible impact of many drugs' intake on vitamin D absorption. However, further pharmacokinetic studies are guaranteed to clarify this outcome. Annweiler et al. [1] study revealed the similar outcomes as the separately studied variable expressed only modest or no association with hypovitaminosis D; however, the combination of all items using the VDSP algorithm effectively indicated severe vitamin D deficiency among elderly patients. In the present study, the VDSP algorithm was not applied. Other reports found that application of a 16-item VDSP tool successfully identified elderly patients with severe vitamin D deficiency ≤25 nmol/L [1], whereas another report observed that the VDSP was effective in identifying mainly vitamin D insufficiency [16].

Also, the added variable of vitamin D supplementation was associated with the vitamin D level as vitamin D deficiency was more reported among participants who did not take vitamin D supplements, whereas vitamin D insufficiency was more reported among those who have taken vitamin D supplements. This finding could be explained by a possible noncompliance of patients on vitamin D supplementation or due to insufficient doses prescribed and/or no intake of calcium to enhance vitamin D absorption. Thus, further studies are recommended to investigate the mentioned possible confounding factors. Notably, identification of adults with severe vitamin D deficiency is particularly important among elderly patients as deficiency/insufficiency of vitamin D occurs gradually. In addition, serum 25(OH)D concentrations below 25 nmol/L suggest severe and chronic hypovitaminosis D, which is associated with severe chronic illness, [20] prolonged hospital stays, [21] and a higher hospital mortality rate [19]. Thus, vitamin D supplementation to elderly adults is considered an essential prescription. Of note, a considerable proportion of elderly patients in this study reported sad emotions, although this was not significantly associated with vitamin D levels, and screening for depression among elderly patients is highly recommended.

Among limitations of the present study, the two important factors which may bias the results were as (a) the dependence upon the physician form of the VDSP, (b) excluding the self-administered questionnaire as self-rating, and (c) single centre patient involvement. Removing these may reflect more accurate outcomes about the health conditions as mentioned in the previous study [22]. Furthermore, the World Health Organization recommends using self-rated tools to rate and monitor individuals' health [23, 24]. Moreover, further studies with a higher sample size and involvement of multiple centres are guaranteed to implement the application of the VDSP algorithm as preferred study design.

## 5. In Conclusion

Our study showed that VDSP tool items with the exception of the number of medications was not associated with serum 25(OH)D levels in elderly people. In addition, vitamin D supplementation was associated with serum 25(OH)D levels among study participants. Further research included a higher participant number (patients on and without vitamin D supplementation) attending different clinics, and the inclusion of the self-rated form of the questionnaire is recommended to strengthen the study's findings.

## Figures and Tables

**Table 1:** Factors associated with vitamin D levels among elderly people, Jeddah.

	Vitamin D level (ng/ml)		*p*-value^*∗*^
	≥50 *N* = 131 *N* (%)	<50 *N* = 204 *N* (%)	
Gender			
Male (*n* = 111)	39 (29.8)	72 (35.3)	
Female (*n* = 224)	92 (70.2)	132 (64.7)	0.295

Age			
Mean ± SD	68.1 ± 7.5	68.2 ± 7.2	0.834^*∗∗*^

Believe of having diseases			
No (*n* = 149)	62 (47.3)	87 (42.6)	
Yes (*n* = 186)	69 (52.7)	117 (57.4)	0.400

Number of drugs taken daily			
<6 (*n* = 137)	39 (29.8)	98 (48.0)	
≥6 (*n* = 198)	92 (70.2)	106 (52.0)	0.001

BMI			
Underweight (*n* = 2)	1 (0.8)	1 (0.5)	
Normal (*n* = 35)	15 (11.5)	20 (9.8)	
Overweight (*n* = 117)	45 (34.3)	72 (35.3)	
Obesity (*n* = 181)	70 (53.4)	111 (54.4)	0.952

History of living alone			
No (*n* = 257)	96 (73.3)	161 (78.9)	
Yes (*n* = 78)	35 (26.7)	43 (21.1)	0.233

Feeling malnourished			
No (*n* = 310)	120 (91.6)	190 (93.1)	
Yes (*n* = 25)	11 (8.4)	14 (6.9)	0.602

Wearing glasses			
No (*n* = 229)	91 (69.5)	138 (67.6)	
Yes (*n* = 106)	40 (30.5)	66 (32.4)	0.727

Regular intake of psychoactive drugs			
No (*n* = 319)	126 (96.2)	193 (94.6)	
Yes (*n* = 16)	5 (3.8)	11 (5.4)	0.509

Feeling sad			
No (*n* = 275)	104 (79.4)	171 (83.8)	
Yes (*n* = 60)	27 (20.6)	33 (6.2)	0.302

Having memory lapses			
No (*n* = 172)	65 (49.6)	107 (52.5)	
Yes (*n* = 163)	66 (50.4)	97 (47.5)	0.613

History of previous fall			
No (*n* = 239)	96 (73.3)	143 (70.1)	
Yes (*n* = 96)	35 (25.7)	61 (29.9)	0.529

Using walking aid			
No (*n* = 223)	81 (61.8)	142 (69.6)	
Yes (*n* = 112)	50 (38.1)	62 (30.4)	0.141

Afraid of fall			
No (*n* = 195)	72 (55.0)	123 (60.3)	
Yes (*n* = 140)	59 (45.0)	81 (39.7)	0.334

History of vertebral fractures			
No (*n* = 290)	113 (86.3)	177 (86.8)	
Yes (*n* = 45)	18 (13.7)	27 (13.2)	0.895

Taking osteoporotic medications			
No (*n* = 299)	114 (87.0)	185 (90.7)	
Yes (*n* = 36)	17 (13.0)	19 (9.3)	0.291

Taking vitamin D supplements			
No (*n* = 194)	55 (42.0)	139 (68.1)	
Yes (*n* = 141)	76 (58.0)	65 (31.9)	<0.001

^*∗*^chi-square test, ^*∗∗*^student *t*-test.

**Table 2:** Factors associated with vitamin D levels among elderly people, Jeddah.

	Vitamin D level				*p* value^*∗*^
	Severe deficiency *N* = 24 *N* (%)	Deficiency *N* = 180 *N* (%)	Insufficiency *N* = 100 *N* (%)	Normal *N* = 31 *N* (%)	
Gender					
Male (*n* = 111)	7 (29.2)	65 (36.1)	34 (34)	5 (16.1)	
Female (*n* = 224)	17 (70.8)	115 (63.9)	66 (66)	26 (33.9)	0.174

Age					
Mean ± SD	68.2 ± 6.5	68.2 ± 7.3	68.1 ± 7.0	68.0 ± 9.1	0.997^*∗∗*^

Believe of having diseases					
No (*n* = 149)	8 (33.3)	79 (43.9)	50 (50)	12 (38.7)	
Yes (*n* = 186)	16 (66.7)	101 (56.1)	50 (50)	19 (61.3)	0.410

Number of drugs taken daily					
<6 (*n* = 137)	12 (50.0)	86 (47.8)	31 (31)	8 (5.8)	
≥6 (*n* = 198)	12 (50.0)	94 (52.2)	69 (69)	23 (11.6)	0.010

BMI					
Underweight (*n* = 2)	0 (0.0)	1 (0.5)	1 (1)	0 (0.0)	
Normal (*n* = 35)	3 (12.5)	17 (9.4)	14 (14)	1 (3.2)	
Overweight (*n* = 117)	6 (25.0)	66 (36.7)	35 (35)	10 (32.3)	
Obesity (*n* = 181)	15 (62.5)	96 (53.3)	50 (50)	20 (64.5)	0.760

History of living alone					
No (*n* = 257)	18 (75.0)	143 (79.4)	76 (76)	20 (64.5)	
Yes (*n* = 78)	6 (25.0)	37 (20.6)	24 (24)	11 (35.5)	0.334

Feeling malnourished					
No (*n* = 310)	24 (100.0)	166 (92.2)	89 (89)	31 (100.0)	
Yes (*n* = 25)	0 (0.0)	14 (7.8)	11 (11)	0 (0.0)	0.099

Wearing glasses					
No (*n* = 229)	18 (75.0)	120 (66.7)	71 (71)	20 (64.5)	
Yes (*n* = 106)	6 (25.0)	60 (33.3)	29 (29)	11 (35.5)	0.738

Regular intake of psychoactive drugs					
No (*n* = 319)	23 (95.8)	170 (94.4)	98 (98)	28 (90.3)	
Yes (*n* = 16)	1 (4.2)	10 (5.6)	2 (2)	3 (9.7)	0.309

Feeling sad					
No (*n* = 275)	20 (83.3)	151 (83.9)	79 (79)	25 (80.6)	
Yes (*n* = 60)	4 (16.7)	29 (16.1)	21 (21)	6 (19.4)	0.773

Having memory lapses					
No (*n* = 172)	14 (58.3)	93 (51.7)	50 (50)	15 (48.4)	
Yes (*n* = 163)	10 (41.7)	87 (48.3)	50 (50)	16 (51.6)	0.883

History of previous fall					
No (*n* = 239)	20 (83.3)	123 (68.3)	72 (72.0)	24 (77.4)	
Yes (*n* = 96)	4 (16.7)	57 (31.7)	28 (28.0)	7 (22.6)	0.382

Using walking aid					
No (*n* = 223)	16 (66.7)	126 (70.0)	63 (63.0)	18 (58.1)	
Yes (*n* = 112)	8 (33.3)	54 (30.0)	37 (37.0)	13 (41.9)	0.470

Afraid of fall					
No (*n* = 195)	16 (66.7)	107 (59.4)	59 (59.0)	13 (41.9)	
Yes (*n* = 140)	8 (33.3)	73 (40.6)	41 (41.0)	18 (58.1)	0.239

History of vertebral fractures					
No (*n* = 290)	23 (95.8)	154 (85.6)	87 (87.0)	26 (83.9)	
Yes (*n* = 45)	1 (4.2)	26 (14.4)	13 (13.0)	5 (16.1)	0.544

Taking osteoporotic medications					
No (*n* = 299)	22 (91.7)	163 (90.6)	87 (87.0)	27 (87.1)	
Yes (*n* = 36)	2 (8.3)	17 (9.4)	13 (13.0)	4 (12.9)	0.767

Taking vitamin D supplements					
No (*n* = 194)	20 (83.3)	119 (66.1)	43 (43.0)	12 (38.7)	
Yes (*n* = 141)	4 (16.7)	61 (33.9)	57 (57.0)	19 (61.3)	<0.001

^*∗*^chi-square test, ^*∗∗*^ANOVA test.

## Data Availability

Raw data are available on request.

## Supplementary Materials

Supplementary Materials The English version of the questionnaire used in this study is provided for the perusal of readers.
