# Correction to “Clinical Identification of Hypovitaminosis D among Elderly Attending Primary Care Centre in Saudi Arabia”

**DOI:** 10.1155/bri/9825279

**Published:** 2025-10-03

A. M. Alzahrani, L. S. Emam, M. S. Alsharif, A. Y. Hakami, and S. S. Aga, “Clinical Identification of Hypovitaminosis D among Elderly Attending Primary Care Centre in Saudi Arabia,” *Biochemistry Research International* 2022 (2022): 6341645, https://doi.org/10.1155/2022/6341645.

In the article titled “Clinical Identification of Hypovitaminosis D among Elderly Attending Primary Care Centre in Saudi Arabia,” author Abdullah M. Alzahrani was affiliated to Department of Primary Health Care, Ministry of National Guard Health Affairs (MNGHA), King Abdulaziz Medical City, Jeddah, Saudi Arabia and Research Office, King Abdullah International Medical Research Centre (KAIMRC), Ministry of National Guard Health Affairs (MNGHA), King Abdulaziz Medical City, Jeddah, Saudi Arabia which is incorrect. The correct affiliations for this author are as follows:

^1^Department of Primary Health Care, Ministry of National Guard Health Affairs (MNGHA), King Abdulaziz Medical City, Jeddah, Saudi Arabia

^2^Research Office, King Abdullah International Medical Research Centre (KAIMRC), Ministry of National Guard Health Affairs (MNGHA), King Abdulaziz Medical City, Jeddah, Saudi Arabia

^3^Department of Basic Medical Sciences, College of Medicine, King Saud Bin Abdul Aziz University for Health Sciences (KSAU-HS), King Abdulaziz Medical City, Jeddah, Saudi Arabia

The corrected list of affiliations is shown in the author information below.

Abdullah M. Alzahrani^1,2,3^, Leena S. Emam^4^, Maryam S. Alsharif^4^, Alqassem Y. Hakami^2,3^, and Syed Sameer Aga^2,3^

^1^Department of Primary Health Care, Ministry of National Guard Health Affairs (MNGHA), King Abdulaziz Medical City, Jeddah, Saudi Arabia

^2^Research Office, King Abdullah International Medical Research Centre (KAIMRC), Ministry of National Guard Health Affairs (MNGHA), King Abdulaziz Medical City, Jeddah, Saudi Arabia

^3^Department of Basic Medical Sciences, College of Medicine, King Saud Bin Abdul Aziz University for Health Sciences (KSAU-HS), King Abdulaziz Medical City, Jeddah, Saudi Arabia

^4^Department of Primary Health Care, Ministry of Health (MOH), Jeddah, Saudi Arabia

We apologize for this error.

