# Supplementary material for: Clinical Identification of Hypovitaminosis D among Elderly Attending Primary Care Centre in Saudi Arabia
Source: Biochem Res Int. 2022 Oct 12;2022:6341645. doi: 10.1155/2022/6341645 (PMC9581707; doi:10.1155/2022/6341645)
Supplement: Supplementary Materials — The English version of the questionnaire used in this study is provided for the perusal of readers. [file 6341645.f1.pdf]

Name: ……………………………………………………

MRN: …………………………

Contact Nr.: …………………………

**Age**	How old are you (in years)? ¦__¦¦__¦¦__¦
**Gender**	Are you…? ☐Female or ☐Male
**General condition**	Do you think you have a lot of diseases? ☐Yes ☐No<br>Number of different drugs daily taken? ¦__¦¦__¦<br>Do you live alone? ☐Yes ☐No
**Nutrition**	What is your weight (in kg)? ¦__¦¦__¦¦__¦, ¦__¦<br>What is your height (in meters)? ¦__¦,¦__¦¦__¦<br>Do you feel malnourished? ☐Yes ☐No
**Vision**	Do you wear glasses? ☐Yes ☐No
**Mood**	Do you regularly take psychoactive drugs? ☐Yes ☐No<br>Do you feel sad? ☐Yes ☐No
**Cognition**	Do you have memory lapses? ☐Yes ☐No
**Gait and falls**	Did you fall in the previous year (at least one fall)? ☐Yes ☐No<br>Do you usually use a walking aid? ☐Yes ☐No<br>Are you afraid of falling? ☐Yes ☐No
**Osteoporosis**	Have you already had vertebral fractures? ☐Yes ☐No<br>Do you usually take osteoporotic medications? ☐Yes ☐No

**Extra question (1)**	Do you currently take Vitamin D tablets/drops or supplements that contain Vitamin D? ☐Yes ☐No

**Extra question (2)**	Questions are answered mainly by? ☐The patient ☐The relative

Vitamin D level: ………………………… nmol/L.

Thank you 😊
